# Evaluation of the Behavior of Carbon Short Fiber Reinforced Concrete (CSFRC) Based on a Multi-Sensory Experimental Investigation and a Numerical Multiscale Approach

**DOI:** 10.3390/ma14227005

**Published:** 2021-11-19

**Authors:** Philipp Lauff, Polina Pugacheva, Matthias Rutzen, Ursula Weiß, Oliver Fischer, Dirk Volkmer, Malte A. Peter, Christian U. Grosse

**Affiliations:** 1 Concrete and Masonry Structures, Technical University of Munich, 80333 Munich, Germany; massivbau@tum.de; 2 Non-Destructive Testing, Technical University of Munich, 81245 Munich, Germany; zfp.zfp@ed.tum.de; 3 Solid State and Materials Chemistry, University of Augsburg, 86152 Augsburg, Germany; matthias.rutzen@physik.uni-augsburg.de; 4 Research Unit Applied Analysis, University of Augsburg, 86135 Augsburg, Germany; ursula.weiss@math.uni-augsburg.de

**Keywords:** carbon short fiber reinforced concrete, multi-scale modeling, multiple microcracking, RVE, 3D-printed concrete, CT material analysis, acoustic emission

## Abstract

Carbon fiber reinforcement used in concrete has become a remarkable alternative to steel fibers. Admixing short fibers to fresh concrete and processing the material with a 3D printer leads to an orientation of fibers and a material with high uniaxial strength properties, which offers an economic use of fibers. To investigate its mechanical behavior, the material is subjected to flexural and tensional tests, combining several measuring techniques. Numerical analysis complements this research. Computed tomography is used with several post-processing algorithms for separating matrix and fibers. This helps to validate fiber alignment and serves as input data for numerical analysis with representative volume elements concatenating real fiber position and orientation with the three-dimensional stress tensor. Flexural and uniaxial tensional tests are performed combining multiple measuring techniques. Next to conventional displacement and strain measuring methods, sound emission analysis, in terms of quantitative event analysis and amplitude appraisal, and also high-resolution digital image correlation accompany the tests. Due to the electrical conductibility of carbon fibers, the material’s resistivity could be measured during testing. All sensors detect the material’s degradation behavior comparably, showing a strain-hardening effect, which results from multiple, yet locally restricted and distributed, microcracks arising in combination with plastic deformation.

## 1. Introduction

The degradation phenomena of fiber-reinforced concrete during fatigue loading is a complex mechanism framed by a combination of the destructive processes fiber rupture, matrix rupture and fiber pull out. Being part of the priority program SPP 2020, this project examines the materials fatigue behavior and crack development of a high strength carbon short fiber reinforced concrete (CSFRC) in an experimental-virtual lab. As preparation for the analysis of complex fatigue tests, this paper describes the investigation of static flexural and tensile tests with multiple sensor types to capture the degradation process and presents convergence studies of accompanying numerical algorithms.

The usage of discontinuous chopped fibers as reinforcement in cementitious composite materials can yield materials with interesting properties but requires careful attention to mixture design and sample preparation. Parameters such as fiber length and aspect ratio, dispersion and wetting behavior of the fibers as well as fiber orientation in the hardened sample have a tremendous impact on the final mechanical properties of the material [1]. Especially fiber length plays an important role, as fibers that are too short cannot effectively transfer tensile loads out of the matrix and thus only contribute to post-cracking toughness by being pulled out of the fiber canal [2].

In contrast, composites in which the (micro-)mechanical behavior of fiber and matrix are tailored to each other show a noticeable increase in tensile strength and a capacity for deformation beyond their linear-elastic limit [3,4]. Stress–strain diagrams for loading scenarios under tension for such composites appear bilinear, with the typical linear-elastic behavior occurring until a first crack is introduced. After this point, deformation behavior known as ‘strain-hardening’ starts to set in. The composite is able to withstand a further increasing load, albeit with noticeably decreased stiffness. This strain-hardening behavior is linked to multiple microcracking appearing in the specimen, as the fibers bridging the cracks thus allow for further transmission of the load. Final failure will only occur once this bridging capacity is eventually exhausted [5,6].

The small and very well controlled width of these microcracks leads to several advantages of these materials when considering durability. The small crack width hinders diffusion of aggressive substances into the material [7] and has even been linked to self-healing capabilities [8,9]. Materials exhibiting such behavior have been described by the umbrella terms ‘strain-hardening cementitious composites’ (SHCC) or ‘engineered cementitious composites’ (ECC). Prior research nearly universally focuses on the use of polymer fibers in their systems. While these fibers provide the composite with an impressive capacity for deformation, the achievable tensile strength is limiting when considering structural use. Prior research by our group shows that composites with enticing strength values (flexural strength above 100 N/mm^2^) can be achieved when using carbon fibers [10,11]. In addition, carbon fibers provide additional benefits in the field of structural health monitoring due to their electric conductivity [12] or fire protection due to better mechanical behavior when exposed to heat [13].

We use a CSFRC with a specifically designed mixture and a fiber content of 1 to 3 vol%. With the help of an additive manufacturing process, in which the admixed carbon fibers are oriented in an almost uniaxial direction, this material gains extremely high flexural and tensile strength and shows a strain-hardening effect [11,14,15]. The use of a 3D concrete printer makes it possible to establish the alignment because of the homogenous material flow inside the printer’s nozzle. The aligned fibers strengthen the concrete extremely in comparison to non-reinforced concrete or a sample with statistically oriented fibers.

The virtual and numerical part of this study consists of a multiscale simulation method. Using X-ray computed tomography (CT) data, the location and orientation of carbon fibers are the basis for structural-oriented modeling in micro-scale. Small representative volume elements (RVE) depict fibers and the cementitious matrix in high definition. Homogenizing these elements for macro-scale simulation leads to global material properties. The question, how changes in structure in micro-scale such as local fiber rupture, fiber–matrix debonding and matrix failure affect macroscopic behavior of fatigue degradation, is one main goal of this research project. Part of this paper is a convergence study of different RVE sizes and numbers of elements.

The data of meso- and macro-scaled experiments delineate the calibration of these calculations. The basic CT data originate from miniature specimens in order to attain images capable of resolving the meso scale. Next to fiber detection, this method helps to discover micro matrix cracks and their development during fatigue testing, as shown in [16]. Here, we especially examine miniature specimens under flexural loading combined with sound wave-based non-destructive testing methods giving an insight into the load-bearing behavior.

On a macroscopic level, locally detached measurement methods accompanied uniaxial tensile tests. Digital image correlation (DIC), in particular, makes a significant contribution to identifying the development of cracks and damage by covering a large measurement range of the specimen’s surface. A new method based on the electrical conductivity of the carbon fibers was also developed and successfully applied to show fiber destruction as a main cause of failure. Furthermore, acoustic emission analysis provides additional insight into the specimen degradation on macro-scale experiments as well. Both sound-based analysis and electrical resistivity measurement enable the detection of damage in the specimen’s inside while DIC gives high-resolution information about microcrack evolution on the surface.

## 2. Raw Material and Sample Preparation

The use of fibers as reinforcement for concrete connects the tensional stress capabilities of the fibers with the compression resistivity of concrete to form a composite material, which can withstand high loads. Most often, fiber reinforcement is either admixed to the fresh concrete as chopped fibers or textile layers consisting of continuous fiber strands are embedded into the molded concrete [17]. The latter has the advantage that most of the fibers are directed uniformly, which reinforces a uniaxial stressed construction in the best way. The arbitrary orientation of short fibers leads to extreme inefficiency, as statistically only 6% of the fibers face in the loading direction within an angle of 20°. If all fibers point in parallel along a beam, they all contribute to the absorption of tensile forces, allowing for a resource-saving and efficient use of short-fiber reinforcement. Studies describing the possibility of achieving an orientation of polymer fibers suggest an extrusion of concrete as a viable way to control fiber alignment [18,19,20,21,22]. The concrete moves uniformly inside the nozzle, which rectifies the admixed fibers along the printed strand’s longitudinal axis. Similarly, an orientation can be achieved by pouring concrete into an oblong mold at one end, letting the concrete flow to the other. Hereby the unidirectional movement leads to fiber alignment parallel to the direction of movement as well [23]. By positioning several extruded strands next to each other, structures build up. This procedure is a 3D printing method called liquid deposition modeling (LDM). It leads to concrete with a high amount of aligned fibers in the direction of nozzle movement [10,14]. By choosing engineered extrusion paths, the possibility to align the fibers according to the principal tensional stresses within the structure exists [24].

Ref. [14] examined the possibility of enhancing the fiber–matrix bond by pretreating the carbon fibers in different ways. This research proposes a thermal de-sizing process under oxidizing conditions that leads to hydrophilization of the fiber surface as oxygen is introduced into the inorganic carbon structure. We adopt this process by heating the carbon fibers to 425 °C and holding the temperature for three hours. Under these conditions, the sizing burns and the fibers’ surface starts to oxidize, increasing its roughness. The positive side effect of increasing the fiber’s hydrophilicity enables the addition and effective dispersion of high fiber amounts of up to 3% by volume to the concrete. This high fiber content requires a high amount of plasticizer to make the mixture suitable for pumping and printing. Table 1 shows the concrete recipe used in this study. Due to the small nozzle of 2 to 4 mm in diameter used for 3D printing, the maximum grain size is 0.5 mm. The water–cement ration is 0.41, including the water in the plasticizer. Carbon fibers are added additionally to the amounts specified.

After production, the specimens are cured at room temperature for one day at 100% RH, set under water for six days and stored for another 21 days with 60% RH.

### 2.1. Preparation of Miniature Bending Beams for Three-Point Bending Tests

Small-sized specimens with dimensions of 60 × 12 × 3 mm were manufactured as described in [16] and subjected to the static three-point bending tests on the universal testing machine Wolpert 10 kN, Ludwigshafen, Germany with a 500 N load cell. The test was conducted in displacement-controlled mode with a constant rate of 0.11 mm/min. For flexural testing, specimens are reinforced with 3 vol.-% carbon fibers (Tenax-J HT C261, Teijin Ltd., Tokyo, Japan).

### 2.2. Macro Scaled Specimen for Uniaxial Tension Tests

Similar to the description in [16], the dry concrete components are admixed in a BECKEL bucket mixer (Schwallungen, Germany), water and plasticizer are added and mixing is continued until nearly all agglomerates are dispersed. Finally, 1 vol.-% carbon fibers (PX35, Zoltek Corporation, Bridgeton, MO, USA) are added and the mixing process is continued shortly for 40 s twice, while in between the bucket’s walls are cleared from dry fibers.

For the production of larger specimens, we developed a 3D concrete printer with a possible working zone of 1.00 × 1.00 × 0.50 m [15]. It consists of three separately controllable axes, which are arranged spatially, to move the printing unit. It is a speed-controlled pneumatic piston pump, which compresses a foil cartridge filled with fresh CSFRC. The nozzle has a quadratic aperture with a lateral length of 4 mm.

The macroscopic specimens are bone-shaped and tested under uniaxial tensional load. Their outermost dimensions are 450 × 100 × 50 mm with a tallied region of a constant width of 50 mm in length in the specimen’s center, leading to a cross section of 50 × 50 mm (Figure 11). In the reducing part, the specimen has a circular outline. Ref. [25] examined the influence of different bone shapes concerning minimum stress peaks due to the specimen’s outline resulting in the aforementioned dimensions. The printing path is parallel to the bone-shaped outline, i.e., circular with increasing radiuses to the vertical centerline to establish the tapering area. Consequently, the carbon fibers, which are oriented in the printed strand’s direction, are aligned to the principal stresses of the specimen under a uniaxial load.

After the printing process has finished, the specimen is molded to gain a more definite surface. This is only necessary to flatten the specimen’s surface to be able to apply measuring equipment. Therefore, the specimen’s prior outline is slightly bigger than desired, as molding compresses the wet concrete. Concomitantly, the specimen’s top is pushed upwards and needs to be cut off after curing with a stone saw Norton CM 501, Wesseling, Germany.

The specimen is glued via connecting steel plates to the machine using a duromer adhesive, MC-DUR 1280 (MC-Bauchemie Müller GmbH & Co. KG, Bottrop, Germany), to establish a stressless clamping at the specimen’s end (Figure 11). Additional steel plates are mounted to the sides in the clamping region to increase friction and to enlarge the area of load transmission. Clamping is preferred to a jointed connection as it leads to a uniaxial loading independent of the distribution of microcracks. The static tests are performed displacement-controlled on a universal testing machine, Roell+Korthaus 100 kN, Ulm, Germany.

## 3. CT Investigation and Numerical Simulation

The basis for numerical analysis is data obtained from X-ray computer tomography. Even the carbon fibers with their very small diameter can be detected to serve as an outline for a FEM-mesh with real fiber distribution.

### 3.1. X-ray Computed Tomography

#### 3.1.1. Measurement

The CT-scans were carried out using a Nanotom M X-ray CT scanner (GE Inspection Technologies LP, Lewistown, PA, USA). An acceleration voltage of 70 kV and a current of 190 µA was set and a full scan consisted of 2000 single images taken at an exposure time of 2000 ms. The scanned sample was prepared with a cross-section of 3.1 mm × 2.2 mm, leading to a voxel size of 1.7 µm. Using phoenix datos|x, the raw data was reconstructed into a 3-dimensional image, converted into RAW-format and imported into ORS Dragonfly 2020.2 (Object Research Systems (ORS) Inc., Montreal, QC, Canada) for segmentation and quantitative analysis.

#### 3.1.2. Segmentation Procedure

As the segmentation of the ‘Tenax-J HT C261′ fiber (Teijin Ltd., Tokyo, Japan; length 3 mm, diameter 7 µm) proved to be too inaccurate to act as a basis for a reliable representative volume element in numerical simulation (see Section 3.2), a sample containing 1 vol% of the larger fiber ‘Kureha Kreca Chop C-103T’ (Kureha Corp., Tokyo, Japan; length: 3 mm, diameter: 18 µm) was segmented instead as a model system, as alignment could also be observed using those fibers.

Carbon fibers are well known to be challenging to segment using conventional X-ray CT. Their small diameter sets strict limits to the dimensions of the sample to be measured, as a similarly small voxel size has to be achieved. In addition, the density of carbon fibers is fairly close to that of the cementitious matrix, making segmentation procedures purely based on the thresholding of grayscale values highly inaccurate. Often, the use of synchrotron CT is seen as necessary [26].

**Figure 1:**
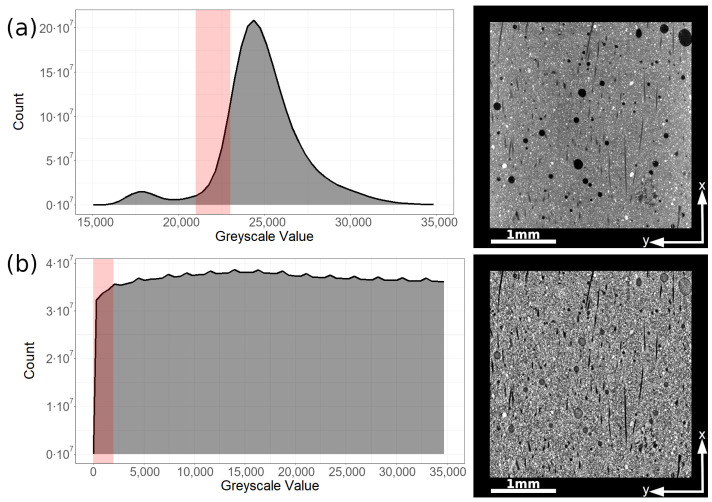
(**a**) Original, unfiltered scan. Fibers vary in grayscale value and overlap with the matrix, making clean segmentation difficult. (**b**) Scan after being filtered through an LHE filter. Fibers appear as monochromatic black with considerably improved contrast to the matrix. The areas marked in red depict the approximate grayscale values corresponding to carbon fibers.

We lay out a segmentation procedure making use of image processing steps as well as geometric parameters of the fibers allowing for successful analysis based on images obtained from a regular laboratory X-ray CT machine. As a first step in segmentation, upper and lower threshold grayscale values have to be defined for the material to be segmented. This is problematic in the original scan image, as not every material present in the composite is represented by a singular separated peak. The grayscale values of our measurements separate broadly into two categories—a first peak at low values (16,000–19,000) corresponding to background and voids as well as a second peak at higher values (21,000–32,000) encompassing all the material present in the sample without further separation (Figure 1a). Coupled with the fact that the fibers themselves are not perfectly uniform in their grayscale value, threshold values derived from the original scan are always highly inaccurate.

Solutions proposed in literature usually rely on segmenting using filters highlighting the low variation of grayscale values within the fiber while there is a discontinuous jump in value at the interface [27]. Further refinement can be carried out using geometric parameters of the desired objects [28]. An algorithm matching the former approach is implemented in ORS Dragonfly called ‘Local Histogram Equalization’ (LHE). The LHE filter is a contrast-enhancing option recalculating a voxel’s grayscale value based on the value of its direct neighbors [29,30]. As a result, the mostly homogenous fibers will appear in a nearly monochromatic black and while the more heterogenic matrix appears in a noisy gray. From the filtered image, a considerably more accurate threshold value can be derived. Distributions of grayscale values and excerpts from the original and filtered scan can be seen in Figure 1. The areas highlighted in red correspond to the approximate values relevant for fiber segmentation.

While the LHE filter allows thresholding methods to work on a principal level, a lot of isolated noise is still picked up alongside the fibers. To allow for further refinement, a connectivity analysis is performed by Dragonfly, identifying each voxel present in the segmentation that connects to at least 6 neighbors as a discrete object. This acts as a basis for the quantitative analysis of the objects and enables further refinement of the segmentation.

**Figure 2:**
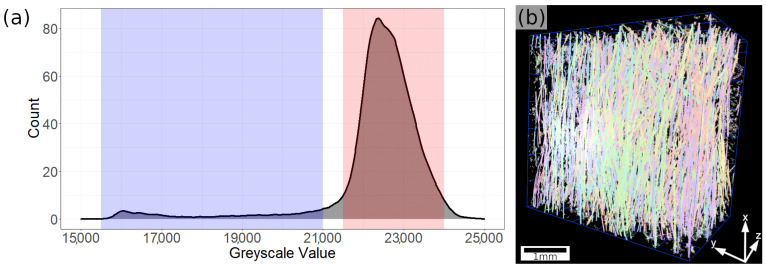
(**a**) Histogram of the objects segmented from the LHE image projected back to the original scan. The separation into discrete peaks highlighted in blue and red allows differentiating between noise and fibers. (**b**) 3-dimensional image of the final segmentation after refinement based on fiber geometry. This segmentation is used for alignment analysis.

The discretized objects can be projected back onto the original reconstructed image and a grayscale value histogram of each voxel intersecting with the segmented objects can be created. As the initial segmentation was based on the filtered image’ s grayscale values and preselected the fibers, this newly created histogram is considerably more significant in terms of material represented in its peaks. As can be seen in Figure 2a, the histogram separates into two regions, with the values below 21,000 mostly representing noise and values above that mark mostly representing fibers. Everything outside the peak highlighted in red is discarded from the segmentation. Final elimination of remaining noise is carried out by a geometric analysis of the remaining objects. Objects with an excessively low volume (<1000 µm^3^) and a high aspect ratio (>0.5) are removed, as they stray too far from proper fiber geometry. The final segmentation seen in Figure 2b is used to quantify the 3-dimensional alignment parameters of the fibers within the sample as well as a basis for a representative volume element (RVE) for modeling purposes.

#### 3.1.3. Alignment Analysis

**Figure 3:**
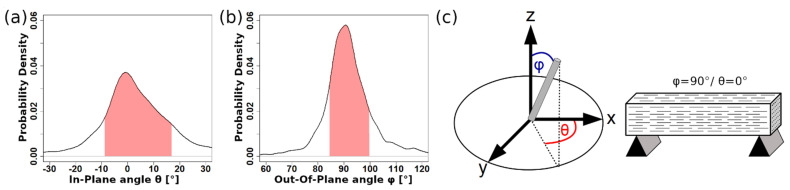
(**a**) Distribution of in-plane alignment angles (*θ*-angles or yaw). (**b**) Distribution of out-of-plane alignment angles (*ϕ*-angles or pitch). (**c**) Depiction of the coordinate system used for definition of angles. The desired alignment angle is pointing in the x-direction, i.e., *θ* = 0° and *ϕ* = 90°. The area shaded in red in (**a**,**b**) denotes a standard deviation of a single *σ*, i.e., 68% of values scattering around the mode of the distribution.

ORS Dragonfly allows for the automatic geometric analysis of segmented objects. Following the steps laid out above enables the export of the 3-dimensional alignment angles of the fibers present in the sample. Their distributions are shown in Figure 3.

As can be seen, both the *θ*- and *ϕ*-angles scatter tightly around specific angles, showing the effectiveness of the alignment during extrusion. In-plane alignment (Figure 3b) occurs mainly around the 0° angle (which coincides with the direction of tensile forces occurring during flexural and tensile testing), deviating at about ±10°. Out-of-plane alignment occurs at the 90°-angle, i.e., fibers lie nearly perfectly horizontally (seen in Figure 3c), scattering at about ±5°.

The results correspond well to other published data on the alignment of samples containing the thinner ‘Tenax-J HT C261′ fiber [10,16], showcasing that the usage of the thicker KrecaChop fiber as a model system still leads to accurate results as far as alignment is concerned.

### 3.2. Numerical Simulations Based on μ-CT Data

The information obtained from CT data provides the basis for the realistic numerical simulation of the mechanical behavior of CSFRC. To enable feasible computation, we propose a two-scale approach that represents both the fiber (micro) scale and the specimen (macro) scale. The approach in means of computational homogenization [31,32,33] requires the determination of a (local) material law in each macroscopic material point by solving cell problems in representative volume elements (RVE) on the fiber scale. In particular, the macroscopic relationship of the deformation gradient ∇u and the stress tensor *σ* is given by mean values of quantities determined in the RVE. This leads to a numerical material law of the form

**Figure 4:**
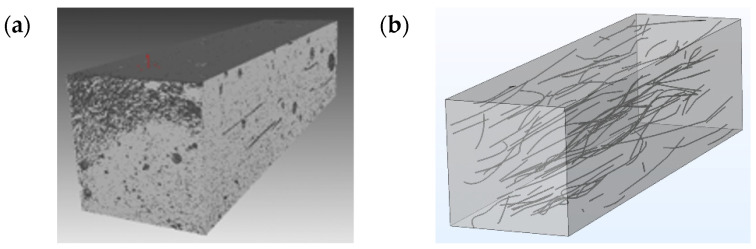
Representative volume element of size 950 μm × 950 μm × 3670 μm: (**a**) from μ-CT data, (**b**) reconstructed in COMSOL Multiphysics^®^. The fibers of the RVE were extracted from μ-CT data and imported into COMSOL Multiphysics^®^ via LiveLink™ [34].

*σ* = f(∇u), (1)

where the mapping f realizes the macro–micro–macro scale transition and represents the local effective material law. Based on own preliminary studies, we neglect aggregates on the fiber scale, meaning the numerical representative volume elements consist only of (homogenized) concrete matrix and fibers. For this purpose, the carbon fibers are identified in typical μ-CT volume cutouts. Specifically, the fiber center curves are marked and imported into the finite element software, where they are cylindrically expanded to the fiber diameter. The aspect ratio of the carbon fibers and the fact that they are arranged almost parallel result in long cubic RVEs. Figure 4 shows an RVE of size 950 μm × 950 μm × 3670 μm obtained from μ-CT data (a) and the reconstructed RVE for CSFRC with fibers of 16.3 μm diameter and 3.1 mm length (b). The full section from which the RVE was extracted was 3.33 mm × 2.44 mm × 3.97 mm and the volume fraction of fibers was 1.08%. The resolution of the μ-CT data is given by the voxel edge length of 1.67 μm, which is sufficient to resolve the fibers of 16.3 μm diameter.

An essential aspect of models based on computational homogenization approaches is the determination of RVEs that are as small as possible and yet representative for the entire material. The efficiency of a numerical model is further influenced by the choice of a mesh size that is just small enough to resolve all relevant mechanisms.

**Figure 5:**
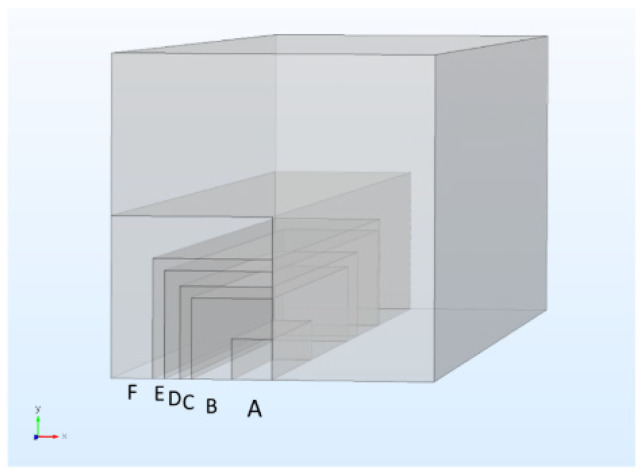
Cut-outs A–F from the overall geometry for convergence studies.

To determine the macroscopic material parameters, convergence studies were performed for different cutouts (A–F) of the underlying overall geometry shown in Figure 5 with size information of the cut-outs in Table 2. The convergence studies in the linear-elastic regime were performed with respect to RVE size and resolution (mesh size) in the framework of a finite element discretization based on quadratic Lagrange elements on simplicial meshes. The material data are set for 7 μm diameter fibers with elastic modulus 230 GPa and Poisson’s ratio 0.2 and concrete matrix with elastic modulus 20 GPa and Poisson’s ratio 0.2. At first the optimal mesh size was determined and, in a second step, the optimal size of the RVE was identified.

#### 3.2.1. Convergence Studies for Resolution

To determine the optimal mesh size, we compare simulation results with the physics-controlled meshes provided in COMSOL Multiphysics^®^ [35] with different element sizes (finer, fine, normal, coarse, coarser and extra coarse) in cutouts B, C, D and E. By this approach the element size parameters, such as maximum and minimum element size in the mesh, are automatically adjusted to changed geometry dimensions.

The calculated homogenized elasticity tensors in Voigt notation serve as the basis for the evaluation. For the calculation of the tensors, periodic boundary conditions were chosen.

**Figure 6:**
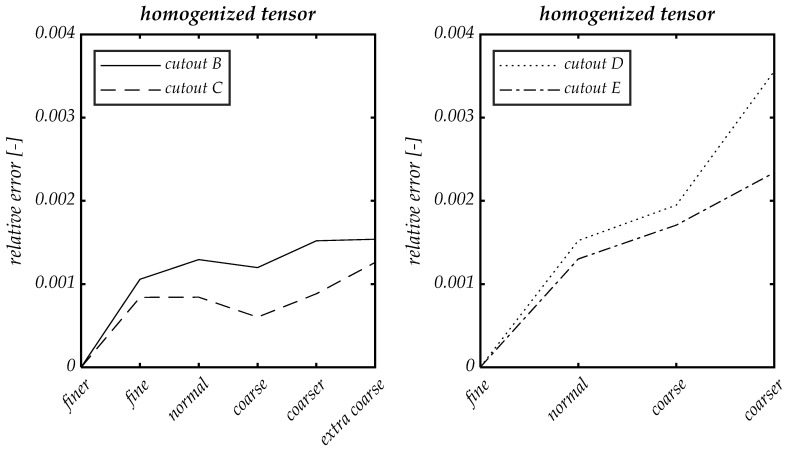
Relative errors of the homogenized tensors for the different element sizes in cut-outs B, C, D and E shown in Figure 5.

The relative errors in relation to the results with the smallest grid size in each case were computed and displayed in Figure 6. For all cutouts, a relative error of the homogenized tensor of less than 0.2% could be achieved with normal element size. Based on this evaluation, a normal mesh size is sufficient to reproduce the corresponding mechanisms. The evaluations of the size of the representative volume element which follows are therefore based on the calculations with normal element size.

#### 3.2.2. Convergence Study on the Size of the RVE

In the sense of an experimental-virtual Lab, it is desirable to determine a numerical representative volume element that is as small as possible in order to keep numerical cost low and yet large enough to be representative.

As before, the homogenized elasticity tensors in Voigt notation serve as the basis for the calculation. In addition to the relative error of the homogenized tensor with respect to the largest section F, we consider the relative errors of the first three diagonal elements of the homogenized tensor in Voigt notation.

**Figure 7:**
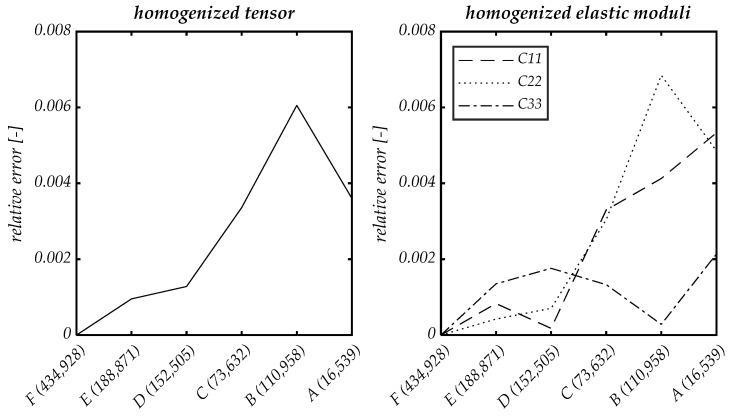
Convergence study of the size of the RVE in cut-outs A–F (number of degrees of freedom in brackets). Relative error of the homogenized tensor (**left**) and relative error of the homogenized elastic moduli in coordinate direction (**right**).

The graph for the convergence study in Figure 7 shows that, already from section D, the relative error of the homogenized tensor and also the individual relative errors of the diagonal components are in the range of the error obtained in the convergence study with respect to mesh size. The convergence studies show that, at least in the linear-elastic range, the microstructure of CSFRC obtained from μ-CT data can be reproduced well with reasonable numerical effort. For further examination, calculations are continued with cutout D and normal element size.

## 4. Flexural Testing of Miniature Bending Beams Combined with AE

Acoustic emission (AE) can be defined as transient elastic waves caused by the release of stored energy due to structural alteration in the material [36]. Compared to other non-destructive monitoring techniques which were developed to examine the structural condition of materials, the AE method has higher sensitivity. Besides detection of the instant of the damage initiation, AE analysis provides particularly an idea of different damage mechanisms, such as matrix cracking, fiber pull-out, or fiber breakage [37,38,39]. Correlating between certain AE features, such as the emitted energy of the AE signal and the cumulative number of AE events, it is feasible to monitor the main stages of crack development with the AE technique [40,41,42,43]. Recently, the AE method has proven to be a meaningful tool for damage evaluation in fiber-reinforced high-performance materials in experimental studies under different loading regimes [44,45,46].

**Figure 8:**
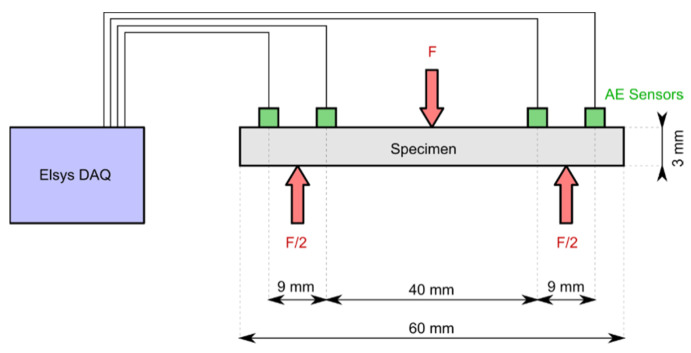
Overview of the experimental setup for static three-point bending tests on miniature beam specimens.

This study considers the concurrent application of the AE technique, stress-time curve analysis and dye penetrant inspection (DP) to assess the damage evolution and fracture behavior in CSFRC during flexural loading. To monitor AE activity, four small-sized passive piezoelectric AE sensors VS700-D (150–800 kHz) were coupled to the edges of the miniature beam specimen (see Figure 8 and Section 2.1) using a hot adhesive. Sensors were connected to the eight-channel acquisition system Elsys, where signals were recorded and digitalized at a 20 MHz sampling rate. Figure 8 provides an overview of the experimental setup.

Analysis of the AE activity is based on three aspects: the interpretation of the time domain features of the signal, the *RMS_AE_* value of the signal and the cumulative number of events. The root-mean-square level of a raw acoustic emission signal, *RMS_AE_*, represents the power of an acoustic signal and can be considered a proxy for the AE energy magnitude [47,48].

**Figure 9:**
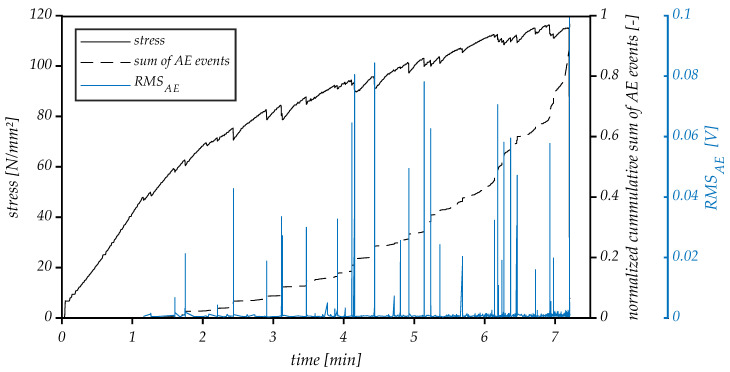
AE signal’s characteristics, RMS_AE and the normalized cumulative sum of AE events and stress curve for the miniature beam specimen.

AE signals were localized in linear geometry by comparing the time delay of the elastic wave between the different sensors. An onset time of AE signal is determined using an automatic picking algorithm based on the Akaike Information Criterion (AIC) [49]. Figure 9 compares the applied stress with the AE signal’s characteristics *RMS_AE_* and normalized cumulative sum of AE events.

The stress development during static, displacement-controlled loading can be characterized by two successive states: an elastic state, where it increases linearly, and a more extended strain-hardening state, where stress continues to rise with a reduced rate relative to the previous period. Failure is very brittle and is characterized by a sudden drop in stress.

Based on the analysis of the *RMS_AE_* and cumulative sum of AE events, two periods of different AE activity which correlate with the specimen’s mechanical behavior are roughly distinguishable. The first one, 0–1.2 min, does not have any AE activity matching the linear elastic behavior in the stress curve, while the second phase, 1.2–7.2 min, has significant, increasing AE activity. During the initial stage, AE activity was absent, suggesting that no damage has yet occurred in the specimen. The first acoustic signals become evident, and the change of the slope of the stress curve marks the onset of microcracking in the concrete matrix, as 40% of the failure load has been reached. At the beginning of the second stage, the recorded AE signal is dominated by low and medium amplitude acoustic emissions, and the normalized cumulative sum of AE events curve increases slowly. With a further increase of the load, the acoustic signals become more pronounced, and higher *RMS_AE_* peaks start to occur. This stage represents the gradual damage accumulation resulting from the stable growth of microcracks in the matrix. This is also visible in the normalized cumulative AE event’s plot as an increase in the gradient of the cumulative events curve. The high activity of AE in the last stage reflects the critical crack growth in the localized zone of maximal stress, ultimately causing the failure of the specimen.

**Figure 10:**
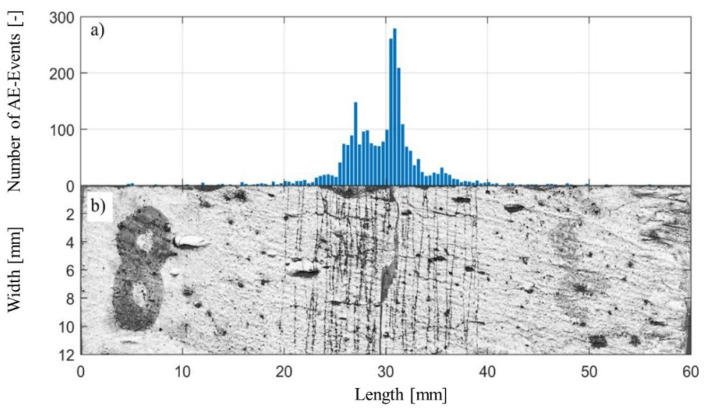
(**a**) Histogram with spatial distribution of the AE events along with the specimen; the y-axis indicates the number of AE events over the length of the specimen, and the width of the individual bins correspond to 0.38 mm; the x-axis corresponds to the length of the specimen; (**b**) shows the crack pattern visible by dye penetrant inspection on the specimen’s bottom.

Figure 10 shows the specimen after failure and the spatial distribution of the AE events. It is evident that the specimen underwent an extensive cracking process before the final failure occurred. Multiple thin cracks opened up in the central area of the specimen on the tensile side (bottom). The eventual failure was initiated by the expansion of the crack located near the centerline of the specimen, slightly closer to the right support region. This conclusion is based on the agreement between the visual inspection of the damage zone and the plot of cumulated locations of the AE events. Most of the AE events originate from the region within 20–40 mm, with the maximum of the events generated near the midpoint of the specimen in the location of the main crack. The spatial AE event distribution reflects the damage zone width and the distinct crack’s actual positions.

## 5. Tensional Testing of Large Bone-Shaped Specimen Combined with DIC, AE and Electrical Resistivity Measurement

As flexural testing is a combination of local compression and tension, it is necessary to investigate the material’s uniaxial behavior as well. In a static flexural analysis, our team found out that using rectangular cross sections, failure occurs not because of collapse in compression zone but due to fiber pull-out or rupture which is shown as a loss of strength in the tensile zone [24]. The fibers in the compression zone are oriented in the direction of stress, meaning they barely influence the material’s compression strength: in compression tests, transverse tension is mostly decisive for the specimen’s failure. However, the fibers are not oriented perpendicular to the transverse tension, so they do not increase the load transfer for this stress direction. Therefore, the compressive behavior is similar to unreinforced concrete and is not part of this paper.

**Figure 11:**
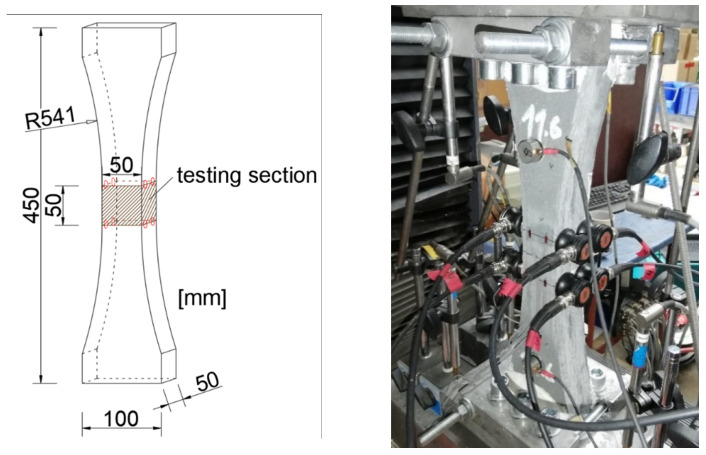
Dimensions of bone-shaped specimen used for the tensile test; the red circles mark the positions of the acoustic emission sensors (**left**). Specimen connected to testing machine with applied sensors (**right**).

For uniaxial tensional tests, macro scaled, bone-shaped specimens (see Figure 11 and Section 2.2) are equipped with various non-destructive measurement methods for the locally differentiated recording of deformations. This enables the detection of the damage process in high precision. In addition to the use of displacement transducers (LVDT) and strain gauges, digital image correlation (DIC) detects strain over a wide area of the specimen’s surface. A single camera (DALSA Genie Nano M4040) is used for a 2D measurement. With its high resolution of 12 MPx, the observation of small deformations and growth of microcracks is possible. The specimens are prepared with white painting and either a randomly black dot pattern or trembling black lines using a rubber stamp.

The degree of damage is also visible by using the non-destructive measurement of electrical resistivity. The high proportion of electrically conductive carbon fibers leads to a well-measurable change in voltage resulting from the connectivity losses of the fibers. This procedure, which is already well established for carbon fiber-reinforced plastics (e.g., [50]), has so far only rarely been applied to concrete specimens [12,51,52]. With the help of a DC voltage source and a series resistor, acquiring the voltage drop at different locations of the test specimen is well suited as a damage detection tool showing qualitative relationships to damaged sections. Depending on the test specimen, the measured resistance values are in the range of a few hundred ohms to a few kilo-ohms.

Furthermore, eight acoustic emission (AE) sensors, V103-RB Olympus (680–1630 kHz), were mounted on the specimen, as shown in Figure 11.

**Figure 12:**
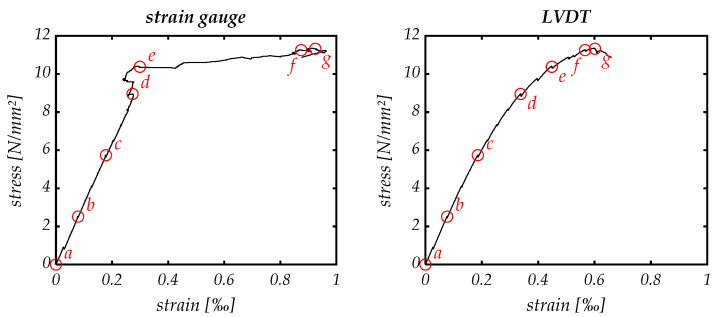
Stress–strain curve of tensional test displaying the mean of four strain gauges (**left**) and three LVDTs (**right**). The red circles (*a*–*g*) mark the stages where pictures were taken for DIC evaluation.

As described above for flexural testing, strain hardening occurs. This effect is also visible in tensional testing, as Figure 12 shows. The strain gauges show linear elastic behavior for a large range of tension. Once a critical point is reached, the strain significantly increases while only little further stress can be applied (point *d*). This point describes the emergence of microcracks at the end of the linear elastic region. Looking at the integral-measuring LVDT sensors, they show similar results; however, the two phases of elastic and plastic deformation cannot be made out as clearly as with strain gauges.

**Figure 13:**
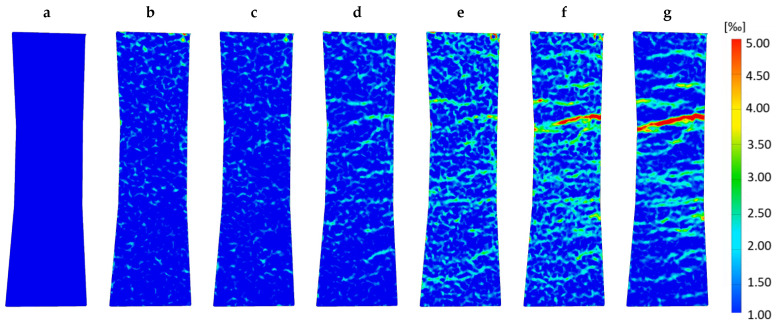
DIC evaluation at stages (**a**–**g**) (see Figure 12) displays strain on the specimen’s surface and shows the formation of multiple microcracks in a wide-spread area. Additionally, the evolution of the crack leading to failure can be observed in pictures (**d**–**g**). Small-sized local strain maxima (light blue) can be subjected to measuring noise.

DIC helps to understand the material’s load-bearing behavior: Not only did several microcracks evolve during the test, but they also extended continuously. Reaching the maximum stress level at points *f* and *g*, Figure 13 reveals a series of densely spaced cracks across the entire width of the specimen. Therefore, the observed increase in strain capacity for the specimen during tension is ensured through the pronounced multiple cracking, which permits the excessive strain energy to be released at failure. One crack spreads out further than the others, resulting in brittle failure. The widening and lengthening of the existing cracks can be observed rather than forming new ones.

The strain gauges measure over a length of 3 mm, which is a lot smaller than the distance between the two cracks visible in Figure 13. They measure at a very specific point in a region of high stress values. In contrast, the LVDTs span over the specimen’s total length measuring also parts with lower stress due to the bone shape leading to a smeared stress–strain relation with smaller overall strain values. The plastic deformations are visible as the gradient decreases continuously starting between points *c* and *d*, concluding that microcracks already appear in a region next to the area where the strain gauges are positioned.

Point *g* marks an instable point where stress decreases while strain still augments. Figure 13 reveals that the final crack already interpenetrates the whole width of the observed specimen’s side, implying that only the failure-causing crack enlarges. It might be possible that the non-observed sides of the specimen are not fully departed yet, and fibers break or are pulled out of the matrix in a zipper principle starting at one point in the cross section. Rupture occurs when the fibers cannot overstretch the crack and withstand the load anymore.

**Figure 14:**
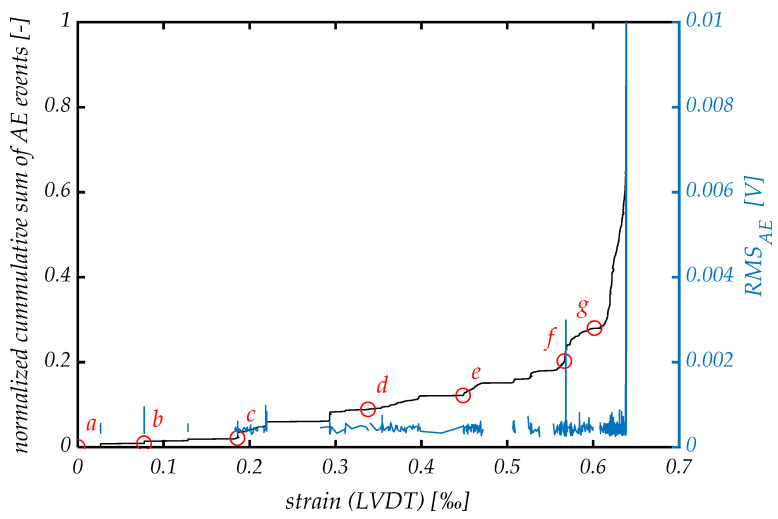
AE signal’s characteristics, the normalized cumulative sum of AE events and *RMS_AE_*. Red circles (*a*–*g*) indicate the different stages in Figure 13.

Looking at the results of the acoustic emission analysis in Figure 14, it is evident that the acoustic emission characteristics significantly varied in CSFRC specimens subjected to different loading conditions. Unlike the three-point bending test, where no acoustic activity was recorded in the linear-elastic stage, the AE signals started appearing at the beginning of monitoring at a low level of stress in the tensile test. As can be seen from the analysis of the *RMS_AE_*, signals do not differ significantly in amplitude throughout the test. In fact, low-amplitude signals dominated the whole loading process, while strong AE signals appeared only in the final phase, in the proximity of the ultimate strength.

A small number of AE signals were recorded during the initial linear elastic stage of the loading between points *a* and *c*, causing a relatively flat growth of the curve of the normalized cumulative sum of AE events. As can be seen in Figure 13, the AE signals in the initial stage are associated with the formation of isolated micro-cracks in the gauge area of the specimen. The acoustic emission method does not discern the formation of the initial crack at the time of transition from the elastic to the plastic deformation stage after point *d*, although it could give some indication of this, namely, a slight increase in the amplitude and density of the AE signal. With the further increase in the stress, the formation of new cracks, their coalescence and propagation are continued, accompanied by moderate acoustic emission activity.

A distinctive feature of the last stage was the strong acoustic emission activity, marked by the appearance of the high-energy AE signals and a sudden increase in the number of cumulative AE events at point *f*. The highest *RMS_AE_* peak amplitude is one order larger than that in previous stages, which coincides with the time of the failure.

**Figure 15:**
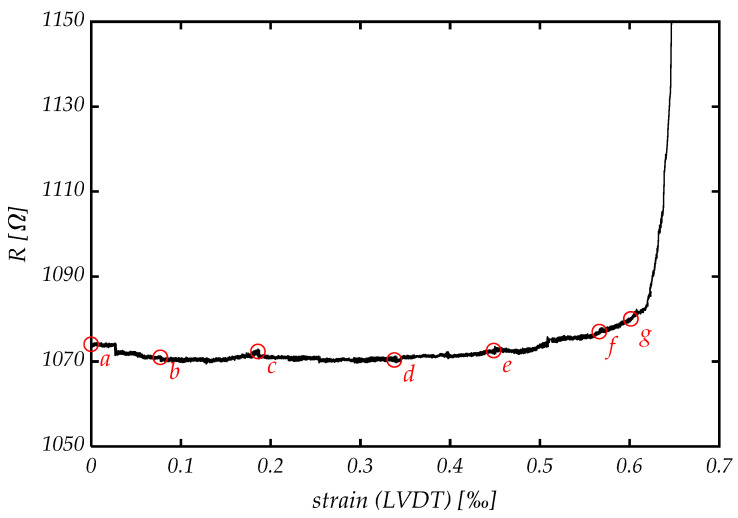
Specimen’s electrical resistance increases especially during plastic deformation after point *d*. Red circles (*a*–*g*) indicate the different stages in Figure 13.

Comparable results can also be obtained from electrical resistivity measurement (Figure 15). The initial specimen’s resistance is about 1070 Ω. While strain grows, the resistivity barely changes until the end of linear elastic behavior is reached at point *d*. From there on, continuous material degradation is detected as resistivity increases exponentially. Especially after reaching the unstable point *g*, the gradient becomes extremely high. The carbon fibers are mainly responsible for good conductivity, concluding that the increase in resistivity is an indication for fiber destruction. This can either be fiber rupture or fiber pullout, although it is not possible to distinguish between either one.

## 6. Conclusions

Carbon short fiber reinforced concrete shows great promise as a material for use in civil engineering structures due to its outstanding performance under tensile load. Results seem very promising regarding both static and dynamic loading. The large amount of oriented fibers in the direction of principal tensile stresses leads to high tensile and flexural capabilities. Hereby, the orientation of the fibers can be achieved by processing the concrete with a 3D printer.

Fiber alignment could successfully be examined using X-ray CT analysis. Several difficulties had to be overcome to be able to separate fibers from the surrounding concrete matrix. This could be achieved by employing various filtering techniques developed for digital image processing and iterating on the result using shape identification algorithms. After segmentation, the retrieved data was analyzed and quantified in view of the fiber’s orientation. Approximately 68% of the fibers are oriented within an angle of ±10° to the desired direction, leading to an economic use of carbon fibers and considerable improvement of tensile and flexural strength. The success of this process poses as a promising starting point for further automation of the as-now still user input-reliant analysis. Further research into alternative segmentation methods (such as those based on deep learning methods) seem especially fruitful for the reliable segmentation of carbon fibers with even smaller diameters.

The CT data are further used for numerical simulation with representative volume elements. For most realistic calculations, fibers and the surrounding matrix are transferred to a small FEM-model. A convergence study using different sizes of the RVE itself and also different mesh sizes shows that in linear-elastic ranges, the material can be represented very well with reasonable calculation efforts.

The performed static tests are a preliminary stage to the analysis of CSFRC under fatigue loading to compare multiple measuring techniques. Both flexural and tensile tests were performed, while the successful combination of acoustic emission analysis, digital image correlation and electrical resistivity measurement as well as conventional displacement and strain measurement lead to a very concise description of the material’s load-bearing behavior. The material shows strain-hardening behavior, which results from multiple, yet locally restricted and distributed, microcracks arising in combination with plastic deformation. Unlike conventional steel fiber reinforced concrete, CSFRC is able to withstand even higher stresses after leaving the linear elastic stage, which classifies the material as a strain-hardening cement-based composite (SHCC). The starting point of matrix degradation was detected comparably by all sensors, though with different intensities and accuracies. While the processes on the macroscale seem relatively clear, we aim to obtain a more complete picture of the CSFRC system by further researching behavior on the single fiber scale. The usage of frequency analyses of acoustic emission datasets is likely to lead to a better understanding of the processes governing final failure, especially the question of whether it is governed by fiber rupture or fiber pullout.

## Figures and Tables

**Table 1:** Concrete recipe for CSFRC with 3D printing ability. Carbon fibers are added additionally to the amounts specified.

Ingredient	Name	Amount
cement	Holcim Sulfo 52, 5R	34.6 wt%
silica fume	Sika Silicol P	21.6 wt%
quartz flour	Quartzwerke SF500	21.6 wt%
quartz sand	Quartzwerke H33	7.6 wt%
water	-	11.6 wt%
plasticizer	BASF Master ACE 460	3.0 wt%
carbon fiber	Tenax-J HT C261 Zoltek PX35	0.82 wt% (per 1 vol.-%)

**Table 2:** Size information of cut-outs.

Cut-Out	Width [μm]	Height [μm]	Depth [μm]
A	119	119	917
B	238	238	1833
C	271	271	2100
D	317	317	2667
E	352	352	2717
F	475	475	3650
total CT-Scan	950	950	3670

## Data Availability

Not applicable.
